# Mitochondrial Adaptation to Mechanical Stress in Cardiac Ageing and Disease

**DOI:** 10.1002/advs.202516736

**Published:** 2025-11-28

**Authors:** Aishwarya Prakash, Thomas Iskratsch

**Affiliations:** ^1^ School of Engineering and Materials Science Queen Mary University of London London E1 4NS UK

**Keywords:** cardiac mechanobiology, heart failure, mechanotransduction, metabolism, mitochondria

## Abstract

Cardiomyocytes are highly specialized cells that depend on a finely tuned interplay between mechanical forces and metabolic activity to sustain continuous contraction throughout life. While the role of mitochondria in supporting cardiac biomechanics through ATP production, calcium buffering, and redox signaling is well established, the reverse relationship, namely how mechanical forces influence mitochondrial behavior, remains comparatively understudied. This review explores the emerging concept of biomechanical feedback on mitochondrial dynamics in cardiomyocytes. Mechanical cues are shown to regulate mitochondrial morphology, positioning, and function via diverse mechanotransduction pathways. Key mechanisms include integrin signaling, stretch‐activated ion channels, and cytoskeletal networks, alongside mechanical stimuli such as cyclic stretch, pressure overload, and shear stress, which modulate mitochondrial fusion/fission processes, membrane potential, calcium handling, and reactive oxygen species production. The implications of these interactions are considered in the context of cardiac pathologies, including hypertrophy, ischemia‐reperfusion injury, and heart failure. By integrating perspectives from mitochondrial biology and cardiac mechanobiology, this review aims to foster interdisciplinary research and inform novel therapeutic approaches for cardiovascular disease.

## Graphical abstract

Mechanical forces shape the heart's energy factories. This review explores how mitochondria in cardiac cells sense and respond to biomechanical stress, altering their structure, positioning, and metabolism. By linking mechanical cues to mitochondrial adaptation, the article highlights pathways driving heart disease and points to new strategies for preserving cardiac energy balance.

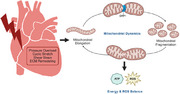

## 1 Introduction

The heart is a biomechanically dynamic organ, continuously subjected to rhythmic cycles of contraction and relaxation that generate mechanical forces essential for blood circulation. At the cellular level, cardiomyocytes, the primary contractile cells of the myocardium must adapt to these mechanical demands while maintaining metabolic homeostasis. This dual requirement is met through a tightly regulated interplay between the cell's structural components and its energy‐producing organelles, particularly mitochondria.

Mitochondria are central to cardiomyocyte function, not only as the primary source of ATP through oxidative phosphorylation but also as regulators of calcium buffering, redox signaling, and apoptotic pathways.^[^ ^1^ ^]^ Their strategic localization within the cell, often aligned with sarcomeres and in close proximity to calcium release units, underscores their integration into the contractile machinery.^[^ ^2^ ^]^ Traditionally, research has focused on how mitochondrial function supports cardiac biomechanics. However, emerging evidence suggests a reciprocal relationship: mechanical forces themselves can modulate mitochondrial behavior, influencing their morphology, distribution, and bioenergetic output.^[^ ^3^ ^]^

This concept of mechanobiological feedback, where mechanical stimuli influence mitochondrial dynamics and function represents an emerging concept in our understanding of cardiac physiology. Mechanotransduction pathways such as integrin signaling, stretch‐activated ion channels, and cytoskeletal networks translate mechanical cues into biochemical signals that regulate mitochondrial fusion, fission, membrane potential, and reactive oxygen species (ROS) production.^[^ ^2^ ^]^ Understanding how mechanical forces shape mitochondrial behavior is particularly relevant in the context of cardiac disease. Conditions such as cardiac hypertrophy, ischemia‐reperfusion injury, and heart failure are characterized by altered mechanical environments and mitochondrial dysfunction.^[^ ^1^ ^]^ Understanding the molecular links between these processes may uncover therapeutic targets and guide interventions to restore mechanical and metabolic balance in heart failure.

This review synthesizes recent research on the bidirectional relationship between mechanical stress and mitochondrial dynamics in cardiomyocytes. We begin by outlining the fundamental aspects of mitochondrial morphology and function in the heart, followed by an exploration of key mechanotransduction pathways. We then examine how specific mechanical forces influence mitochondrial structure and activity, and how cytoskeletal elements mediate these effects. Finally, we discuss the implications of these interactions in cardiac pathology and highlight emerging directions for future research.

## 2 Mitochondrial Dynamics in Cardiomyocytes

**Figure 1:**
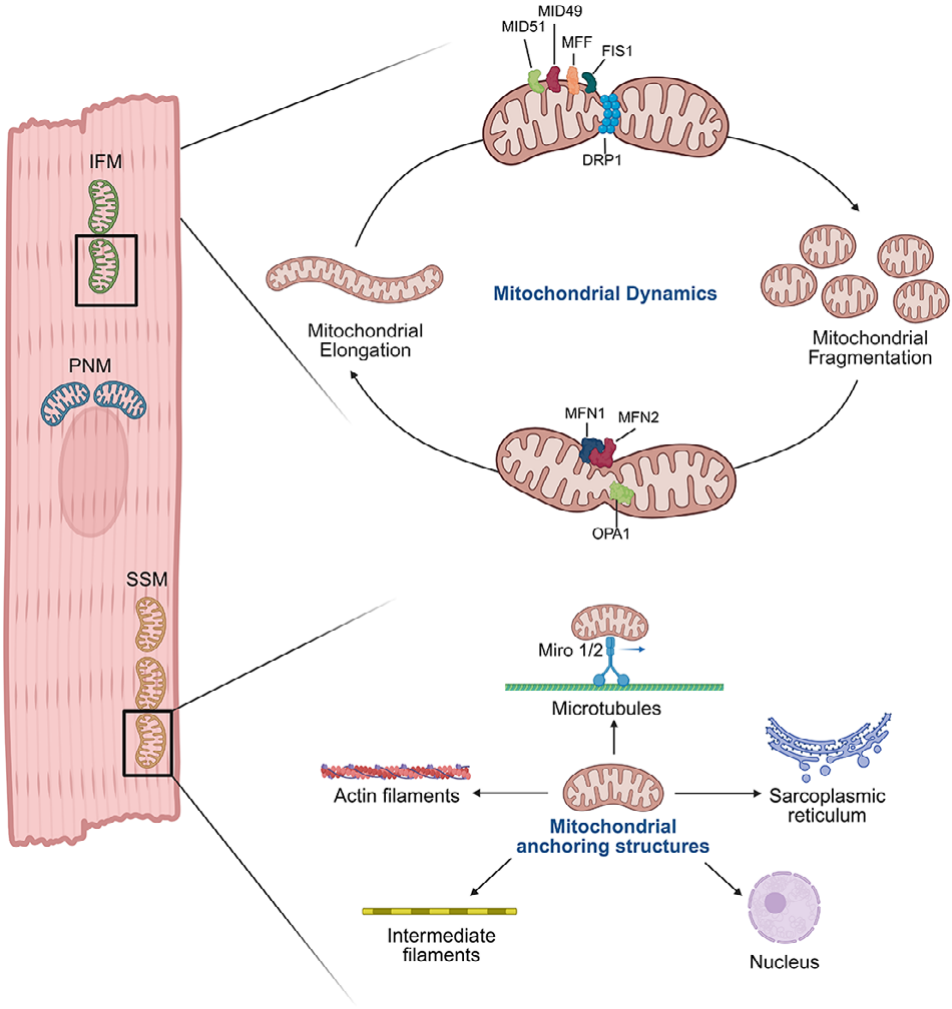
Mitochondrial Dynamics and Spatial Organization in Cardiomyocytes. This schematic illustrates the three distinct mitochondrial populations in cardiomyocytes‐subsarcolemmal (SSM), interfibrillar (IFM), and perinuclear (PNM), and highlights their structural connections to cytoskeletal elements and organelles. SSM mitochondria are located beneath the plasma membrane and support ion channel function; IFM mitochondria are aligned between sarcomeres to supply ATP for contraction; and PNM mitochondria cluster around the nucleus to regulate transcription and biogenesis. The diagram also depicts mitochondrial fusion and fission processes, mediated by mitofusin1/2 (MFN1/2), optic atrophy 1 (OPA1), and dynamin‐related protein (DRP1), mitochondrial dynamics protein of 49 and 51 (MiD49, MiD51), mitochondrial fission 1 protein (Fis1), mitochondrial fission factor (MFF) respectively which maintain mitochondrial quality control and adapt morphology in response to mechanical and metabolic stress. Actin filaments, microtubules, and intermediate filaments maintain mitochondrial positioning through adaptor proteins such as mitochondrial Rho GTPases 1/2 (Miro1/2) Tethering to the sarcoplasmic reticulum (via MFN2) and nucleus supports calcium exchange and transcriptional regulation. These anchoring networks enable mitochondria to resist mechanical strain and preserve energy distribution during contraction.

Mitochondria in cardiomyocytes exist as a dynamic network constantly undergoing morphological and positional changes in response to cellular demands. These dynamics are governed by mitochondrial fusion and fission which are essential for maintaining mitochondrial function, quality control, and adaptation to stress (**Figure** **1**).^[^ ^4^ ^]^

### 2.1 Mitochondrial Morphology: Fusion, Fission, and Network Formation

Mitochondrial fusion facilitates the merging of individual mitochondrial networks to form a larger one. This process allows the mixing of mitochondrial contents including mitochondrial DNA, proteins, and metabolites which help maintain mitochondrial function and mitigate damage.^[^ ^5^ ^]^ This process is primarily mediated by mitofusin 1 and 2 (MFN1/2) on the outer mitochondrial membrane and optic atrophy 1 (OPA1) on the inner membrane. Conversely, mitochondrial fission facilitates the segregation of damaged mitochondria and is essential for mitophagy and cell division. Dynamin‐related protein 1 (DRP1) is the key regulator of fission, recruited from the cytosol to the mitochondrial surface where it interacts with mitochondrial dynamics protein of 49 and 51 (MiD49, MiD51), mitochondrial fission 1 protein (Fis1) and mitochondrial fission factor (MFF) which then constricts and divides the organelle. In cardiomyocytes, the balance between fusion and fission is tightly regulated and critical for maintaining mitochondrial and cellular homeostasis. Disruption of this balance has been implicated in various cardiac pathologies, including ischemia‐reperfusion injury and heart failure.

### 2.2 Spatial Organization: Alignment with Sarcomeres and Subcellular Localization

In physiological conditions, the cardiomyocyte mitochondria are highly organized within the cell, typically arranged in three distinct populations: subsarcolemmal (SSM), interfibrillar (IFM), and perinuclear (PNM, Figure 1).^[^ ^6^ ^]^ The SSM mitochondria are found beneath the cell membrane and are involved in ion channel function and signaling pathways. The PNM mitochondria are located near the nucleus and are involved in nuclear function, mitochondrial biogenesis, and play a role in regulating gene expression related to mitochondrial function. The IFM mitochondria, which are the most abundant, are aligned between sarcomeres to ensure efficient ATP delivery to the contractile apparatus. This spatial arrangement is not random but is maintained by cytoskeletal elements and tethering proteins that anchor mitochondria in place.^[^ ^7^, ^8^ ^]^

Such precise localization supports the high energy demands of cardiac contraction and facilitates localized calcium signaling. It also suggests that mitochondrial positioning is not only a structural feature but a functional adaptation to the mechanical and metabolic environment of the cardiomyocyte.

### 2.3 Functional Implications: ATP Production, Calcium Buffering, and ROS Signaling

Mitochondria are the primary source of ATP in cardiomyocytes, producing over 90% of the cell's energy through oxidative phosphorylation.^[^ ^9^ ^]^ This energy is essential for sustaining the continuous contractile activity of the heart. In addition to energy production, mitochondria play a pivotal role in buffering cytosolic calcium, particularly during excitation‐contraction coupling.^[^ ^10^ ^]^ They take up calcium via the mitochondrial calcium uniporter (MCU), which helps regulate both mitochondrial metabolism and cytosolic calcium transients.^[^ ^11^ ^]^

Furthermore, mitochondria are major sources and targets of reactive oxygen species (ROS). While low levels of ROS serve as signaling molecules, excessive ROS production can lead to oxidative damage, mitochondrial dysfunction, and cell death.^[^ ^12^, ^13^ ^]^ The dynamic behavior of mitochondria, through fusion, fission, and movement, modulates these functions and allows the cell to adapt to changing physiological and pathological conditions.

### 2.4 Mitochondrial Biogenesis, Degradation, and Mitophagy under Mechanical Stress

Beyond fusion, fission, and spatial organization, mitochondrial homeostasis in cardiomyocytes relies on biogenesis and quality control mechanisms such as mitophagy. Biogenesis is primarily regulated by transcriptional coactivators including PGC‐1α, NRF1/2, and TFAM, which drive mitochondrial DNA replication and respiratory protein synthesis. Mechanical cues such as cyclic stretch have been shown to upregulate PGC‐1α and TFAM expression, enhancing mitochondrial mass and oxidative phosphorylation capacity.^[^ ^14^ ^]^ Conversely, pathological mechanical stress, including pressure overload, can suppress biogenesis through Hippo‐YAP/TAZ signaling, leading to reduced expression of mitochondrial genes and impaired energy production.^[^ ^15^ ^]^

Mitophagy serves as a critical degradation pathway to remove damaged mitochondria and maintain cellular homeostasis. Mechanical stress activates mitophagy via PINK1‐Parkin signaling, particularly under conditions of pressure overload and ischemia‐reperfusion injury, where mitochondrial depolarization and ROS accumulation act as triggers.^[^ ^16^, ^17^ ^]^ ECM remodeling and altered stiffness can also influence mitophagic activity, linking extracellular mechanical cues to mitochondrial quality control.^[^ ^18^, ^19^, ^20^ ^]^ These processes are integrated with mechanotransduction pathways such as RhoA/ROCK and MAPK, which modulate DRP1 activity and mitochondrial fragmentation, thereby coupling cytoskeletal tension to mitochondrial turnover.^[^ ^16^, ^21^, ^22^, ^23^ ^]^

Together, biogenesis and mitophagy dynamically respond to mechanical environments, ensuring mitochondrial adaptability during physiological load and stress.

## 3 Mechanotransduction Pathways and Mechanosensors in Cardiomyocytes

Cardiomyocytes are uniquely adapted to sense and respond to mechanical stimuli generated by the rhythmic contraction and relaxation of the heart. These mechanical forces ranging from extracellular matrix stiffness, passive tension, cyclic stretch, shear stress to pressure overload are transduced into intracellular biochemical signals through a network of mechanosensors and signaling pathways.^[^ ^24^, ^25^ ^]^ This process, known as mechanotransduction, plays a pivotal role in regulating mitochondrial dynamics, function, and overall cellular homeostasis (**Table** **1**). Mitochondrial fusion and fission are tightly regulated processes that respond to mechanical cues, and their dysregulation is increasingly recognized as a contributor to cardiac pathology. Understanding the molecular components of mechanotransduction is essential for elucidating how mechanical stress contributes to mitochondrial adaptation and dysfunction in cardiovascular ageing and disease. Here we highlight first the role of the different mechanical stimuli on cardiomyocyte metabolism.

**Table 1:** Summary of mechanical stimuli and their effects on mitochondrial dynamics in cardiomyocytes. This table synthesizes key mechanical inputs encountered by cardiomyocytes, the associated mechanotransduction pathways, mitochondrial responses, and resulting pathological outcomes. It highlights how distinct mechanical environments such as cyclic stretch, pressure overload, shear stress, and extracellular matrix (ECM) stiffness, modulate mitochondrial morphology and function through specific signaling cascades, contributing to either adaptive remodeling or disease progression.

Mechanical Stimulus	Mechanotransduction Pathways	Mitochondrial Response	Pathological Outcome	Refs.
Cyclic Stretch	Integrins, Ion Channels, Cytoskeleton	↑ Biogenesis (PGC1‐α, TFAM), ↑ ATP ↑ Fission (DRP1)	Adaptive or Fragmentation	[14, 27]
Pressure Overload	YAP/TAZ, RhoA–ROCK, ATF4	↓ MFN1/DRP1, ↑ ROS, ↓ NADPH	Hypertrophy, Remodeling	[17, 63, 109, 113]
Shear Stress	ERK5, MCU, Mitophagy	↑ Calcium influx, ↑ ROS, ↑ KLF2	Protective or Dysfunction	[28, 29]
ECM Stiffness	Integrins, SETD3, Cytoskeleton	Altered morphology, ↑ Respiration	Remodeling, Aging	[18, 20, 90]
Stretch‐Activated Channels	Piezo1, TRPV4, MCU	↑ Calcium uptake, mPTP opening	Apoptosis, Arrhythmia	[82, 119, 131, 132]

### 3.1 Cyclic Stretch and Mitochondrial Remodeling

Mechanical stretch is a fundamental biomechanical stimulus in cardiomyocytes, mimicking the rhythmic expansion and contraction of the heart. Recent studies have shown that cyclic stretch can induce both adaptive and maladaptive changes in mitochondrial morphology and function. Mitochondria undergo physical deformation during the cardiac mechanical cycle, particularly at the Z‐disc and intercalated disc regions.^[^ ^26^ ^]^ These deformations may influence mitochondrial alignment and cristae structure, potentially affecting energy distribution and calcium buffering during contraction. Moreover, cyclic stretch applied to HL‐1 murine cardiomyocytes significantly increased the expression of mitochondrial biogenesis‐related genes such as PGC1‐α, TFAM, and ERRα, as well as oxidative phosphorylation‐related proteins including PHB1 and NDUFS1.^[^ ^14^ ^]^ This was accompanied by increased mitochondrial mass and ATP production, suggesting that cyclic stretch can enhance mitochondrial function under controlled conditions. Further evidence from Vejandla et al. revealed that mutations in the sarcomeric protein nebulette, which disrupt its interaction with desmin and focal adhesions, impair mitochondrial integrity under mechanical strain.^[^ ^27^ ^]^ In neonatal rat cardiomyocytes subjected to cyclic stretch, mutant NEBL failed to redistribute properly, leading to mitochondrial disorganization and reduced tolerance to biomechanical stress.

Together, these studies highlight the dual nature of cyclic stretch: while it can promote mitochondrial biogenesis and function in healthy cells, it may also exacerbate mitochondrial fragmentation and dysfunction in disease contexts or under genetic perturbation.

### 3.2 Shear Stress and Mitochondrial Adaptation

Shear stress is a critical mechanical cue in cardiovascular physiology, yet its role in regulating mitochondrial dynamics in cardiomyocytes remains underexplored. While most studies focus on endothelial cells, emerging evidence suggests that shear stress may also influence mitochondrial function in cardiac tissues through conserved mechanotransduction pathways.

Low‐shear stress preserved mitochondrial function and cell viability in porcine cardiomyocytes in a microfluidic model of ischemia/reperfusion injury.^[^ ^28^ ^]^ This suggests that even subtle mechanical flow can modulate mitochondrial resilience under pathological conditions.

More mechanistic insights come from Coon et al. who showed that laminar shear stress (LSS) activates a dual pathway in endothelial cells involving both MEKK2/3–MEK5–ERK5 signaling and mitochondrial metabolism.^[^ ^29^ ^]^ LSS‐induced mitochondrial calcium influx and ROS production triggered mitophagy, which amplified ERK5 activation and promoted expression of the anti‐inflammatory transcription factor KLF2. Disruption of mitochondrial calcium uptake or mitophagy (via EMRE or PINK1 knockout) impaired ERK5 activation and KLF2 induction, highlighting the importance of mitochondrial remodeling in shear stress signaling.

Although these findings were derived from endothelial models, the mechanistic parallels such as calcium‐dependent mitochondrial activation and mitophagy are likely relevant to cardiomyocytes, especially in engineered heart tissues exposed to flow. However, direct evidence in cardiac cells remains limited. Addressing this gap will be critical for understanding how mechanical flow contributes to cardiac metabolic adaptation and resilience in disease.

### 3.3 Role of the ECM and ECM Mechanics

The extracellular matrix (ECM) provides not only structural support but also mechanical and biochemical cues that regulate cardiomyocyte function. ECM derived from adult human hearts promotes improved mitochondrial and metabolic maturation in human iPSC‐derived cardiomyocytes.^[^ ^30^ ^]^ This includes mitochondrial elongation, increased membrane potential, and enhanced oxidative phosphorylation, demonstrating that native cardiac ECM contains biochemical cues essential for mitochondrial development and function. However, also changes to the ECM in ageing and disease have the potential to negatively influence mitochondrial behavior.

Lyra‐Leite et al. demonstrated in a range of studies that both ECM rigidity and cardiomyocyte shape independently regulate mitochondrial network architecture.^[^ ^18^, ^19^, ^20^ ^]^ Using different engineered substrates with varying stiffness and aspect ratios, they showed that matrix stiffness, composition as well as cell geometry influence mitochondrial surface area, volume, distribution, respiration and glycolytic activity, highlighting the importance of mechanical cues in maintaining mitochondrial health during cardiac development and disease.

ECM remodeling during myocardial stress can further activate mitophagic pathways.^[^ ^31^ ^]^ Mitochondrial degradation via mitophagy is tightly coupled to matrix metabolism, suggesting that ECM turnover influences mitochondrial quality control mechanisms. This connection may be particularly relevant in ischemia‐reperfusion injury and heart failure, where both ECM and mitochondrial integrity are compromised.

## 4 Overview of Mechanosensors

The mechanical stimuli are sensed through different cardiomyocyte mechanosensors, specialized cellular structures that detect mechanical changes in the extracellular and intracellular environment.^[^ ^32^ ^]^ Cardiomyocyte mechanosensing has been previously reviewed elsewhere,^[^ ^33^ ^]^ hence we will provide here only a brief overview of the primary cardiomyocyte mechanosensors.

### 4.1 Integrins

These transmembrane receptors form focal adhesion complexes that link the ECM to the actin cytoskeleton. Different integrins bind to the extracellular matrix ligands, with laminin binding α6β1 and α7β1 being the most abundant in healthy adult cardiomyocytes, while fibronectin binding integrins are upregulated in heart disease, with implications on cardiomyocyte‐ECM coupling and mechanical sensing behavior.^[^ ^33^, ^34^ ^]^ Upon mechanical stimulation, integrins activate intracellular signaling cascades such as focal adhesion kinase (FAK), Src family kinases, and Rho GTPases, which influence mitochondrial morphology and bioenergetics.^[^ ^35^, ^36^ ^]^ Integrin‐mediated signaling modulates Rho GTPase activity, and through that cytoskeletal tension,^[^ ^32^, ^37^ ^]^ which can further affect mitochondrial positioning and trafficking.^[^ ^38^ ^]^

### 4.2 Stretch‐activated ion channels

Stretch‐activated ion channels are key mediators of mechanotransduction in cardiomyocytes, linking mechanical stress to intracellular signaling pathways that regulate mitochondrial function and cellular adaptation. Among these, Piezo1, TRPV4, and TREK‐1 have been implicated in modulating calcium and potassium fluxes in response to biomechanical stimuli:

Piezo1 is a mechanosensitive cation channel that is upregulated in response to pressure overload and localizes to the T‐tubules and intercalated discs of cardiomyocytes.^[^ ^39^ ^]^ Its activation by mechanical stretch or the agonist Yoda1 induces calcium influx and activates calcineurin and calpain signaling, promoting hypertrophic gene expression and cardiomyocyte enlargement. Cardiac‐specific Piezo1 knockout attenuates transverse aortic constriction (TAC)‐induced hypertrophy, fibrosis, and contractile dysfunction, confirming its role in pathological remodeling. Independently, Piezo1 has also been shown to mediate stretch‐induced calcium sparks and ROS production via a Rac1–NOX2–RyR2 axis, linking mechanical stress to mitochondrial redox signaling.^[^ ^40^ ^]^

TRPV4, a calcium‐permeable channel activated by osmotic and mechanical stress, is upregulated in aged cardiomyocytes and localizes to microtubules and the T‐tubule network. Its activation enhances sarcoplasmic reticulum calcium content and ryanodine receptor mediated release, augmenting contractility. However, sustained TRPV4 activity leads to mitochondrial calcium overload and cell death during ischemia–reperfusion, effects that are mitigated by TRPV4 inhibition.^[^ ^41^ ^]^

TREK‐1, a two‐pore domain potassium channel, is activated by ATP through a dual MAPK pathway involving p38 and ERK1/2, leading to cPLA2 translocation and arachidonic acid release. This activation stabilizes mitochondrial membrane potential and reduces calcium‐induced mitochondrial permeability transition pore (mPTP) opening, suggesting a protective role during mechanical stress.^[^ ^42^ ^]^

### 4.3 Other Mechanosensors

Cell‐cell junctions such as desmosomes, adherens junctions, and gap junctions are concentrated at the intercalated disc (ICD), a specialized structure that connects adjacent cardiomyocytes.^[^ ^43^ ^]^ The ICD not only facilitates electrical and mechanical coupling but also serves as a hub for mechanotransduction. Proteins such as N‐cadherin, plakoglobin, and connexin‐43 (Cx43) respond to mechanical stress and can influence intracellular signaling cascades that affect mitochondrial function.^[^ ^44^ ^]^ Studies using a mouse model of dilated cardiomyopathy (DCM) characterized by the knockout of the muscle LIM protein, have found a lack of mitochondria near the ICD, suggesting the dysregulation of mitochondrial organization during growth contributing to heart disease.^[^ ^45^ ^]^ Ultrastructural analyses in a clinical pathological comparative study have revealed that mitochondrial distribution and morphology near the ICD are altered in various cardiac pathologies, suggesting a functional link between cell–cell adhesion and mitochondrial adaptation.^[^ ^46^ ^]^

Cx43 is not only localized at the sarcolemma but also at cardiomyocyte mitochondria, and that its mitochondrial content increases significantly following ischemic preconditioning (IP). This mitochondrial pool of Cx43 may contribute to cardioprotection by modulating mPTP opening and reducing reperfusion injury.^[^ ^47^ ^]^ Their findings suggest that mechanical stress, such as that induced during IP, can enhance mitochondrial Cx43 localization, potentially linking ICD mechanotransduction to mitochondrial resilience. Transmembrane protein 65 (Tmem65) is a further regulator of ICD structure and function. Tmem65 interacts with Cx43 and the sodium channel β1 subunit, and its knockdown in mice leads to disrupted ICD architecture, mis‐localization of Cx43 and Nav1.5, and impaired cardiac conduction. Notably, Tmem65 deficiency also resulted in reduced mitochondrial clustering near the ICD and altered mitochondrial–ICD contact, suggesting a mechanistic link between ICD integrity and mitochondrial positioning and function.^[^ ^48^ ^]^ A summary of key mechanosensors in cardiomyocytes, their localization, mechanical inputs, and downstream effects on mitochondrial function is provided in **Table** **2**.

**Table 2:** Mechanosensors in cardiomyocytes and their influence on mitochondrial function. Major mechanosensors in cardiomyocytes include integrins, stretch‐activated ion channels, cytoskeletal elements, and intercellular junctions. This table outlines their cellular localization, the mechanical stimuli they detect, and the downstream effects on mitochondrial morphology, positioning, and bioenergetics. These mechanosensors form the basis of mechanotransduction pathways that integrate mechanical stress with mitochondrial adaptation, contributing to cardiac homeostasis or pathology depending on context.

Mechanosensor	Location	Mechanical Input	Downstream Effect on Mitochondria	Refs.
Integrins	Cell membrane	ECM stiffness	FAK/Src activation, cytoskeletal tension	[35, 36, 131]
Piezo1/TRPV4	Membrane	Stretch	↑ Calcium influx → MCU → ROS, ATP	[118, 132]
Cytoskeleton	Intracellular	Tension	Mitochondrial anchoring, trafficking	[97, 101, 133]
Intercalated Disc	Cell‐cell junction	Load transfer	Mitochondrial redistribution, signaling	[43, 44, 45, 46]

## 5 Signal Transduction Cascades

Mechanical stimuli activate a variety of intracellular signaling pathways that converge on mitochondrial function. These cascades integrate mechanical inputs with mitochondrial responses, enabling cardiomyocytes to adapt to biomechanical demands.

### 5.1 MAPK (Mitogen‐Activated Protein Kinase) Pathways

Mechanical stress activates ERK1/2, JNK, and p38 MAPKs, which regulate mitochondrial gene expression, apoptosis, and oxidative stress responses.^[^ ^49^, ^50^ ^]^ The mechanical stress induced in myocardial ischemia reperfusion injury (IRI) has known to activate p38 MAPK which in turn induces cellular death.^[^ ^51^, ^52^ ^]^ Recent studies in a model of Parkinsons disease have linked the activation of this pathway to the direct phosphorylation of DRP1 at the Ser616 residue thereby activating mitochondrial fission which potentially links this mechanical stress induced signaling pathway in modulating mitochondrial dynamics.^[^ ^53^ ^]^

### 5.2 RhoA/ROCK Pathway

RhoA belonging to the Rho‐family of small GTPases along with its downstream effector, the Rho‐associated coiled‐coil containing serine‐threonine kinase (ROCK) mediate actomyosin contractile force and cell survival in response to mechanical stimuli.^[^ ^21^, ^22^, ^23^ ^]^ Studies in the past few years have shed light on the role of this signaling pathway in phosphorylating the Ser616 residue of DRP1, in turn activating mitochondrial fission and cellular death in the setting of IRI.^[^ ^54^, ^117^ ^]^ Apart from observing this phenomenon in cardiomyocytes, studies in pulmonary endothelial cells have also shown that nitration mediated activation of RhoA stimulated mitochondrial fission via ROCK‐dependant phosphorylation of DRP1 at the Ser616 residue.^[^ ^16^ ^]^

Other studies have expanded the role of RhoA/ROCK signaling to mitochondrial quality control and metabolic adaptation.  In neonatal rat ventricular myocytes and in vivo mouse models, RhoA activation was found to stabilize PINK1 protein at mitochondria, promoting Parkin recruitment and mitophagy.^[^ ^55^ ^]^ Beyond the cardiovascular system, RhoA/ROCK signaling was found to regulate DRP1‐mediated mitochondrial fission during collective cell migration in Drosophila border cells.^[^ ^56^ ^]^ RhoA and ROCK promoted DRP1 recruitment to mitochondria, enhancing ATP production and actomyosin‐driven protrusive behavior. This study revealed a direct link between cytoskeletal tension and mitochondrial dynamics, suggesting that RhoA/ROCK coordinates energy production with mechanical output during migration.

### 5.3 Hippo‐YAP/TAZ Pathway

The Hippo pathway modulates the activity of the YAP/TAZ protein complex which act as transcription factors in pathways impacting cardiac homeostasis.^[^ ^57^ ^]^ Various mechanical forces including ECM rigidity, shear tension, and cell contractility have been demonstrated to modulate the activation of YAP/TAZ.^[^ ^58^ ^]^ A more recent article by Wu et al. linked the activation of the Hippo pathway in mediating mitochondrial damage by repressing mitochondrial genes that promote the development of DCM.^[^ ^15^ ^]^ Specifically, Hippo signaling inhibited the transcriptional activity of YAP/TAZ, leading to downregulation of genes involved in mitochondrial biogenesis, oxidative phosphorylation, and fusion/fission dynamics. This repression resulted in mitochondrial fragmentation, reduced ATP production, and increased susceptibility to cardiomyocyte apoptosis, ultimately contributing to ventricular dilation and contractile dysfunction.

These signaling pathways are highly interconnected. This intricate network allows cardiomyocytes to fine‐tune mitochondrial responses to mechanical stress but also creates vulnerability to dysregulation in disease states.

## 6 Mechanical Regulation of Mitochondrial Form and Function

Mechanical stress not only reshapes mitochondrial morphology but also profoundly influences mitochondrial function. Cardiomyocytes adapt to biomechanical cues by modulating mitochondrial membrane potential, calcium uptake, ROS production, and ATP synthesis‐ processes that are tightly integrated with cellular signaling and metabolic reprogramming.

### 6.1 Mechanosensitive Regulation of Fragmentation and Fission

Biomechanical cues alter mitochondrial morphology by modulating the activity of key fusion and fission proteins—DRP1, OPA1, and mitofusins (MFN1/2). These proteins act as molecular switches that integrate mechanical cues into mitochondrial structural responses, thereby influencing cardiomyocyte survival, energy metabolism, and stress adaptation.

DRP1 is a cytosolic GTPase that translocate to the outer mitochondrial membrane upon activation, where it oligomerizes and constricts mitochondria to facilitate fission.  Evidence from Chua et al. showed that mechanical stretch downregulated microRNA‐499 (miR‐499), which normally suppresses calcineurin A (CnA), a phosphatase that activates DRP1 through dephosphorylation.^[^ ^59^ ^]^ This miR‐499–CnA–DRP1 axis promotes mitochondrial fission and apoptosis in stretched cardiomyocytes, linking mechanical stress to mitochondrial fragmentation via transcriptional regulation. Additionally, Chang et al. used phosphoproteomic profiling in pressure‐overloaded mouse hearts to identify DRP1 as a key modulator of cardiac hypertrophy, with increased phosphorylation at Ser616 correlating with mitochondrial fragmentation and pathological remodeling.^[^ ^60^ ^]^

OPA1, located on the inner mitochondrial membrane, regulates both fusion and cristae architecture. Mechanical stress can disrupt OPA1 processing, leading to cristae disorganization and impaired oxidative phosphorylation. Although direct mechanosensitive regulation of OPA1 remains less defined, studies suggest that altered mitochondrial membrane potential and calcium flux, both influenced by mechanical cues, can affect OPA1 cleavage and function.^[^ ^61^, ^62^ ^]^

MFN1 and MFN2 mediate outer membrane fusion and also participate in tethering mitochondria to the endoplasmic reticulum (ER), facilitating calcium exchange and metabolic coordination. Pressure overload represses MFN1 transcription, contributing to mitochondrial fragmentation and hypertrophic remodeling.^[^ ^63^ ^]^ Additionally, cytoskeletal tension transmitted via integrins and actomyosin networks may influence MFN‐mediated fusion by altering mitochondrial positioning and membrane curvature.

The coordinated regulation of DRP1, OPA1, and MFNs under mechanical stress reflects a broader integration of mechanotransduction pathways with mitochondrial quality control. For example, calcium influx through stretch‐activated channels can modulate DRP1 activity via calcineurin‐dependent dephosphorylation,^[^ ^64^ ^]^ while ROS generated during mechanical strain may cause oxidative damage to mitochondrial proteins, impairing fusion/fission.^[^ ^65^, ^66^ ^]^ These interactions underscore the dynamic responsiveness of mitochondrial morphology to mechanical environments and highlight potential therapeutic targets for restoring mitochondrial balance in cardiac disease.

Additional evidence from vascular studies reinforces the impact of mechanical stress on mitochondrial remodeling. Hydrostatic pressure combined with hypoxia synergistically upregulates mitochondrial HSP60 and induces its partial cytosolic redistribution in pulmonary arterial smooth muscle cells, promoting phenotypic switching and inflammatory signaling.^[^ ^67^ ^]^ Similarly, cyclic stretch in pulmonary endothelial cells elevates mitochondrial ROS and stabilizes HIF‐1α, linking mechanical strain to redox signaling and dysfunction.^[^ ^68^ ^]^ Beyond magnitude, the variability of mechanical forces also plays a critical role: physiological fluctuations in cyclic stretch maintain mitochondrial networks near a percolation threshold, optimizing ATP production and minimizing ROS, whereas pathological variability disrupts this balance and drives fragmentation through dysregulation of MFN1/2, OPA1, and DRP1.^[^ ^69^ ^]^ These findings complement cardiac data showing DRP1 activation and MFN1 repression under pressure overload^[^ ^60^, ^63^ ^]^ underscoring that mitochondria act as mechanoresponsive organelles integrating tension cues with structural and metabolic adaptation.

While mitochondrial positioning in mature cardiomyocytes is highly constrained by sarcomeric architecture,^[^ ^70^ ^]^ mechanical stress can still elicit dynamic responses at different timescales. Acute mechanical stimuli, such as transient stretch or pressure changes, primarily modulate mitochondrial function altering membrane potential, calcium uptake, and ATP output without major structural rearrangements. These rapid adaptations ensure energy homeostasis during fluctuating load. In contrast, chronic mechanical stress, as seen in pressure overload or sustained hypertrophy, engages mitochondrial quality control pathways and fusion/fission machinery, leading to fragmentation, altered cristae architecture, and impaired oxidative phosphorylation. This temporal distinction aligns with evidence that DRP1 activation^[^ ^60^ ^]^ and MFN1 repression^[^ ^63^ ^]^ occur under prolonged overload, whereas early responses involve metabolic reprogramming and ROS signaling^[^ ^65^, ^66^ ^]^ rather than large‐scale organelle redistribution. Future studies should systematically compare short‐ and long‐term mechanical loading to clarify these adaptive versus maladaptive transitions.

### 6.2 ETC, Mitochondrial Membrane Potential and ATP Output

The electron transport chain (ETC) creates the mitochondrial membrane potential (ΔΨm) and drives mitochondrial ATP production in cardiomyocytes, enabling continuous contractile activity.

Under physiological conditions, mechanical cues promote ETC integrity, and ΔΨm is tightly maintained to support oxidative phosphorylation and ATP production.^[^ ^71^ ^]^ Protective factors such as PHB2 help stabilize inner mitochondrial membrane proteins, preserving ETC structure and function under mechanical stress, such as pressure overload conditions.^[^ ^72^ ^]^

Conversely, pathological mechanical stress can impair cristae architecture and disrupt ETC function. Sustained mechanical stretch in neonatal cardiomyocytes reduced ΔΨm via mPTP opening, triggering apoptosis.^[^ ^73^ ^]^ Exposing endothelial cells to a physiological shear stress of 10 dyn cm^−2^ showed increased ΔΨm by 30%, while high shear stress resembling pathological conditions (60 dyn cm^−2^) decreased ΔΨm by 20%.^[^ ^74^ ^]^ Deficiency of the complex I enzyme NDUFS1 in pressure‐overloaded hearts also aggravated ΔΨm dysfunction and increased mitochondrial ROS production.^[^ ^75^ ^]^

Importantly, interventions that restore mechanical balance or reinforce mitochondrial architecture can help preserve ΔΨm. Therefore, therapeutic strategies aimed at reinforcing ETC resilience are gaining traction. Activation of estrogen‐related receptors (ERRs) enhances fatty acid oxidation and ETC activity improved cardiac function in pressure overload models.^[^ ^76^ ^]^ Additionally, ECM‐derived scaffolds and cytoskeletal stabilizers have been shown to preserve ETC integrity and ATP production under mechanical stress.^[^ ^19^, ^30^ ^]^

Overall, ΔΨm serves as a sensitive readout of mitochondrial adaptation to mechanical cues. Its regulation reflects the interplay between structural integrity, energy demand, and stress signaling, making it a central target for understanding and modulating cardiac bioenergetics under mechanical load.

### 6.3 Calcium Uptake and mPTP Sensitivity

Calcium signaling is central to cardiomyocyte function, and mitochondria play a key role in buffering cytosolic calcium during excitation–contraction coupling. Mechanical stretch may initially impact the ER, a highly deformable organelle that serves as the primary calcium reservoir. ER deformation under biomechanical load can alter calcium release dynamics through IP3R and RyR channels, thereby influencing mitochondrial calcium uptake at mitochondria‐associated membranes (MAMs). This mechanosensitive ER–mitochondria crosstalk integrates excitation–contraction coupling with metabolic adaptation, linking early ionic responses to downstream mitochondrial function and stress signaling.^[^ ^77^, ^78^ ^]^ However, excessive calcium influx, often triggered by mechanical strain or dysregulated ion channel activity, can lead to mitochondrial calcium overload. In failing cardiomyocytes, mitochondrial calcium overload contributes to arrhythmogenesis and metabolic remodeling, linking impaired calcium handling to electrical instability.^[^ ^79^ ^]^

Mechanical stress‐induced calcium influx is taken up by mitochondria via the MCU complex. This calcium uptake regulates metabolic enzyme activity, ROS production, and mitochondrial membrane potential.  While earlier studies questioned the relevance of MCU1/2 gating in the heart,^[^ ^80^ ^]^ recent genetic and biochemical evidence confirms their critical role.^[^ ^81^ ^]^ MCU1 deletion nearly abolishes calcium uptake, while MCU2 loss causes a moderate reduction, highlighting their gatekeeping function and protective role against calcium overload and mitochondrial injury.^[^ ^81^ ^]^

Additionally, extracellular chloride depletion has been shown to protect cardiomyocytes from hypoxia/reoxygenation injury by attenuating mPTP opening, preserving mitochondrial membrane potential, and reducing ROS generation.^[^ ^82^ ^]^ ‐ highlighting the interplay between ionic homeostasis, calcium signaling, and mitochondrial function

Nevertheless, impaired mitochondrial calcium uptake can also stem from other sources. In early diabetic cardiomyopathy, disrupted calcium transfer is linked to mitochondria‐associated membranes (MAMs), regions of the endoplasmic reticulum which are tethered to the mitochondria. Dia et al. showed that reduced IP3R–Grp75–VDAC complex formation limits calcium flux from the ER to mitochondria, compromising ATP production and contractility despite intact MCU and MCU1 levels.^[^ ^77^ ^]^ Additionally, mechanical stress influences calcium influx through stretch‐activated ion channels such as Piezo1 and TRPV4, which modulate cytosolic calcium levels and indirectly affect mitochondrial uptake. Similarly, mechanical stimuli at the sarcolemmal level can alter mitochondrial calcium microdomains, triggering maladaptive responses including mPTP opening and ROS production.^[^ ^78^ ^]^

### 6.4 ROS Production and Redox Signaling

ROS are key mediators of mitochondrial signaling and stress responses in cardiomyocytes. While physiological levels of ROS contribute to adaptive signaling, mechanical stress can elevate ROS production, leading to oxidative damage and mitochondrial dysfunction.

Mitochondria generate ROS primarily at complexes I and III of the electron transport chain (ETC).^[^ ^83^ ^]^ Mechanical instability, such as loss of myosin 19‐mediated tethering, disrupts cristae architecture and mitochondrial membrane potential, thereby impairing oxidative phosphorylation and potentially increasing electron leakage and ROS production under mechanical strain.^[^ ^84^ ^]^ This oxidative burden impairs mitochondrial proteins, lipids, and DNA, compromising ATP production and promoting apoptosis.

Several protective mechanisms have been identified that counteract ROS elevation under mechanical stress. Melusin, a muscle‐specific chaperone, localizes to mitochondria and modulates fatty acid oxidation. Melusin interacts with the mitochondrial trifunctional protein to limit ROS generation during pressure overload and doxorubicin‐induced stress, thereby preserving mitochondrial function and cardiac resilience.^[^ ^85^ ^]^ Mechanical stimuli such as stretch and angiotensin II also amplify ROS production and apoptosis. All‐trans retinoic acid inhibits stretch‐induced ROS generation and mitochondrial dysfunction by upregulating antioxidant defenses such as superoxide dismutase 2 and modulating mitochondrial membrane potential.^[^ ^86^ ^]^

In disease contexts, mechanical stress further exacerbates ROS‐mediated damage. Jung et al. revealed that osmotic shock in dystrophic cardiomyocytes triggers excessive cytosolic and mitochondrial Ca^2^⁺ signals, leading to ROS production and collapse of membrane potential which is a precursor to cell death.^[^ ^87^ ^]^

Ageing enhances these effects. Aged cardiomyocytes exhibit elevated ROS and altered Zn^2^⁺ transporter expression, contributing to oxidative stress and contractile dysfunction.^[^ ^88^ ^]^ Therapeutic targeting of ROS in aged rat hearts using MitoTEMPO, a mitochondria‐targeted antioxidant, reduced ROS levels and reversed structural and functional impairments, including mitochondrial clustering and lysosomal accumulation.^[^ ^89^ ^]^

These studies underscore the dual role of ROS as both a sensor and effector of mechanical stress. Understanding how biomechanical cues regulate mitochondrial redox balance is essential for developing interventions that mitigate oxidative damage and preserve cardiac function.

## 7 Cytoskeletal‐Mitochondrial Interactions

Cytoskeletal components act as primary mechanosensors and first responders to mechanical stress, including stretch and shear forces. Microtubules, actin filaments, and intermediate filaments rapidly remodel under these conditions, influencing mitochondrial positioning, motility, and morphology. The cytoskeleton is a central mediator of mechanical force transmission in cardiomyocytes and plays a critical role in organizing mitochondrial architecture and function. Composed of actin filaments, microtubules, and intermediate filaments, the cytoskeletal network not only maintains cellular integrity but also facilitates mitochondrial trafficking, anchoring, and morphological adaptation in response to mechanical stress (Figure 1). This mechanosensitive behavior integrates cytoskeletal dynamics with mitochondrial adaptation, highlighting that cytoskeletal integrity is a prerequisite for mitochondrial resilience under biomechanical load.

### 7.1 Actin Filaments and Mitochondrial Function

Actin filaments serve as dynamic scaffolds that regulate mitochondrial morphology, positioning, and function in cardiomyocytes, particularly under mechanical stress.

SETD3, a mechanosensitive actin methyltransferase, promotes F‐actin polymerization around mitochondria, enhancing their elongation, movement, and oxidative phosphorylation. Its expression is regulated by extracellular matrix stiffness, linking mechanical cues to mitochondrial dynamics.^[^ ^90^ ^]^ The sarcomeric actin crosslinker α‐actinin‐2 mutations disrupted mitochondrial structure and gene expression through the MRTF‐SRF pathway,^[^ ^91^ ^]^ where monomeric actin sequesters myocardin‐related transcription factor A (MRTFA) and impairs its nuclear localization. This mechanosensitive transcriptional axis links actin dynamics to mitochondrial biogenesis and cardiomyocyte development. βII spectrin, an actin‐associated protein was further demonstrated to be a critical regulator of mitochondrial homeostasis and regulated the translocation and activity of mitochondrial complex I subunit NDUFS1.^[^ ^92^ ^]^ Its deletion in cardiomyocytes leads to impaired respiration and worsened ischemia/reperfusion injury, highlighting its role in maintaining mitochondrial integrity under stress.

Together, these studies highlight the multifaceted role of actin and actin binding proteins in integrating mechanical signals with mitochondrial structure and function. Through direct tethering, or indirect regulation, actin serves as a mechanosensitive scaffold that enables cardiomyocytes to adapt their metabolic machinery to biomechanical demands.

### 7.2 Microtubules and Organelle Trafficking

Microtubules are essential for mitochondrial transport, positioning, and inter‐organelle communication in cardiomyocytes. They serve as tracks for motor proteins such as kinesin and dynein, enabling dynamic mitochondrial responses to mechanical and metabolic stress.

Recent studies have highlighted the role of FBP2, a bifunctional enzyme, in regulating mitochondrial motility and microtubule stability. Its oligomeric state influences mitochondrial movement and cytoskeletal organization, suggesting a dual role in energy metabolism and structural integrity.^[^ ^93^ ^]^ During cardiomyocyte maturation, centrosome reorganization, a process involving microtubule organizing centers, has been linked to mitochondrial and sarcomeric remodeling. Disruption of this process in infantile dilated cardiomyopathy impairs mitochondrial structure and function, underscoring the developmental importance of microtubule‐mediated organelle positioning.^[^ ^94^ ^]^ Microtubule‐dependent trafficking is also mediated by Miro1, an outer mitochondrial membrane protein that anchors mitochondria to motor proteins. In diabetic cardiomyopathy, Miro1 dysfunction leads to mitochondrial arrest and impaired energy distribution, contributing to contractile dysfunction.^[^ ^95^ ^]^

Mechanical stress and disease states such as atrial fibrillation disrupt sarcoplasmic reticulum–mitochondrial contacts (SRMCs), which rely on microtubule integrity and MFN2 tethering. Loss of SRMCs impairs calcium signaling and mitochondrial function, promoting arrhythmogenesis and metabolic imbalance.^[^ ^96^ ^]^ Additionally, microtubule remodeling has been shown to influence mitochondrial fission and fusion dynamics. Jones and Naylor emphasized that microtubules not only guide mitochondrial movement but also regulate organelle shape and division, integrating cytoskeletal cues with mitochondrial morphology across diverse cell types.^[^ ^97^ ^]^

### 7.3 Intermediate Filaments and Structural Anchoring

Intermediate filaments, particularly desmin, play a central role in maintaining mitochondrial organization and mechanical resilience in cardiomyocytes. They form a lattice that connects mitochondria to sarcomeres, Z‐discs, and intercalated discs, ensuring efficient energy distribution and structural integrity under mechanical load.

Desmin interacts closely with αB‐crystallin, a small heat shock protein that stabilizes intermediate filaments and protects mitochondria from stress‐induced damage. Diokmetzidou et al. showed that loss of desmin or αB‐crystallin disrupts mitochondrial morphology, impairs respiratory function, and increases susceptibility to apoptosis, highlighting their cooperative role in maintaining mitochondrial homeostasis.^[^ ^98^ ^]^ Mutations in desmin lead to early signs of architectural and biomechanical failure in cardiomyocytes. Diermeier et al. demonstrated that desmin‐mutant myofibers exhibit mitochondrial fragmentation, reduced elasticity, and impaired contractility, linking cytoskeletal disruption to metabolic dysfunction.^[^ ^99^ ^]^ Desmin's anchoring function is mediated by plectin, a cytolinker protein that targets intermediate filaments to Z‐discs and costameres. Konieczny et al. showed that distinct plectin isoforms tether mitochondria to these structural hubs, preserving myofiber integrity and mitochondrial network architecture.^[^ ^100^ ^]^

Beyond structural support, intermediate filaments also participate in signaling. Marzetti et al. emphasized the crosstalk between mitochondria and the cytoskeleton, noting that desmin filaments influence mitochondrial dynamics, calcium handling, and oxidative stress responses.^[^ ^101^ ^]^

### 7.4 Cytoskeletal Adaptors and Mitochondrial Anchorage

Cytoskeletal adaptor proteins serve as critical linkers between mitochondria and the structural cytoskeleton, enabling precise mitochondrial positioning, anchoring, and mechanosensitive regulation in cardiomyocytes.

Plectin, a versatile cytolinker, anchors mitochondria to intermediate filaments and Z‐discs. Specific isoforms of plectin localize to mitochondria and costameres, stabilizing mitochondrial networks and preserving myofibrillar integrity. Loss of plectin disrupts mitochondrial alignment and contributes to contractile dysfunction, particularly under mechanical stress.^[^ ^100^ ^]^ Ankyrins, particularly ankyrin‐B, are emerging as key adaptors that tether mitochondria to the sarcoplasmic reticulum and plasma membrane domains. Mutations in ankyrin‐B disrupt mitochondrial calcium handling and contribute to arrhythmogenic cardiomyopathy, highlighting the importance of adaptor‐mediated spatial organization for mitochondrial function.^[^ ^102^ ^]^ Additionally, mitochondrial Rho GTPases (Miro1/2), which link mitochondria to kinesin motors, also act as adaptors that sense calcium and regulate mitochondrial motility. Their dysfunction in hypertrophic and pressure overloaded hearts leads to mitochondrial arrest and impaired energy distribution, reinforcing the role of adaptor proteins in integrating mechanical and metabolic signals.^[^ ^103^, ^104^ ^]^

Lastly, syntaphilin, although more extensively studied in neurons, has been shown to immobilize mitochondria by anchoring them to microtubules.^[^ ^105^ ^]^ While its role in cardiomyocytes remains less defined, its mechanistic function suggests a potential role in regulating mitochondrial distribution during mechanical overload or metabolic demand.

## 8 Mechanosensitive Crosstalk Between Mitochondria and the Nucleus

Mitochondrial function is not only shaped by mechanical stress but also actively communicates with the nucleus to regulate gene expression and metabolic adaptation. Mitochondrial dysfunction can trigger retrograde signaling to the nucleus. Loss of membrane potential, accumulation of mitochondrial DNA (mtDNA) fragments, and altered metabolite levels (e.g., NAD+/NADH, succinate) act as stress signals that reshape nuclear transcription.^[^ ^106^, ^107^, ^108^ ^]^ Activation of ATF4 in response to redox imbalance and amino acid depletion promotes expression of genes involved in serine/glycine metabolism and antioxidant defense, restoring mitochondrial homeostasis under mechanical stress.^[^ ^109^ ^]^

The nucleus itself functions as a mechanosensitive organelle that integrates mechanical cues to regulate gene expression and metabolic responses. Cardiomyocyte nuclei undergo rhythmic deformation during contraction, and their structural components including the nuclear envelope, lamina, and chromatin, play active roles in sensing and transducing mechanical signals and this can be influenced by mitochondria directly and indirectly. Mitochondrial clustering around the nucleus, triggered by bafilomycin A1, leads to nuclear deformation, increased stiffness, and DNA damage, independent of oxidative stress.^[^ ^110^ ^]^ These changes are accompanied by altered Lamin A/C morphology and phosphorylation, suggesting that mechanical compression from organelle repositioning can compromise nuclear integrity. Mechanical stress also modulates mitochondrial biogenesis through transcriptional regulation. For instance YAP/TAZ translocate into the nucleus in response to nuclear strain, regulating, amongst others genes involved in mitochondrial biogenesis, glycolysis, and lipid metabolism which are pathways critical for cardiomyocyte adaptation under stress.^[^ ^111^, ^112^ ^]^ Mechanical stress activates nuclear signaling through mechanotransduction pathways involving integrins, cytoskeletal networks, and the linker of nucleoskeleton and cytoskeleton (LINC) complex, which transmit forces to the nuclear envelope and chromatin. These mechanical cues modulate transcriptional regulators such as YAP/TAZ and ATF4, driving gene programs that influence mitochondrial biogenesis, oxidative phosphorylation, and stress responses. This direct mechanosensitive nuclear control complements retrograde mitochondrial signaling, forming a bidirectional axis that integrates biomechanical load with metabolic adaptation.^[^ ^111^, ^112^ ^]^

This bidirectional crosstalk enables cardiomyocytes to fine‐tune their transcriptional programs in response to biomechanical cues and mitochondrial status, particularly during stress and disease.

## 9 Pathophysiological Implications

**Figure 2:**
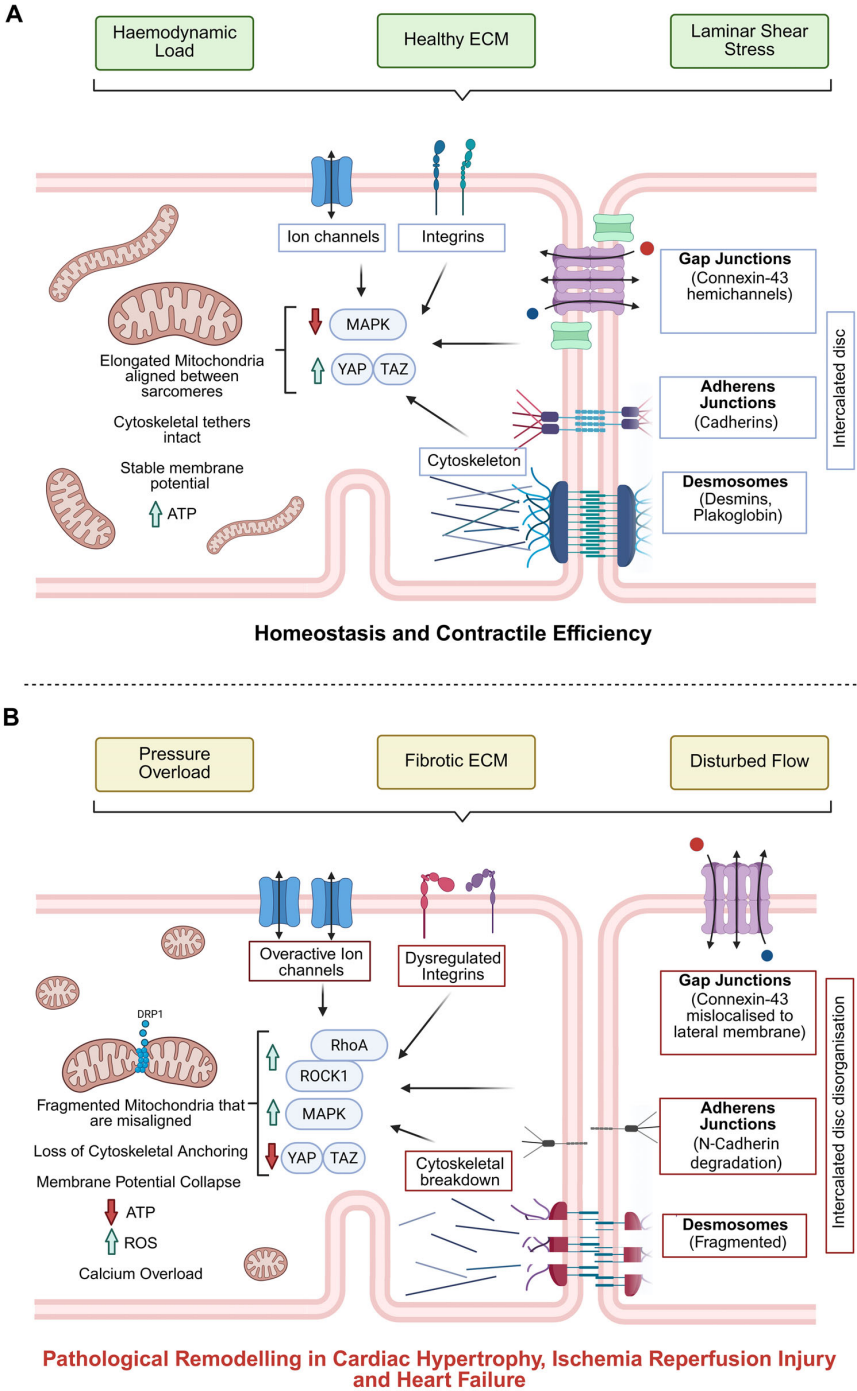
Mechanical Stress‐driven Mitochondrial Remodeling in Cardiomyocytes: Physiological versus Pathological States. Mechanical stress results in bidirectional effects of on mitochondrial structure and function in cardiomyocytes. The top panel (A) depicts physiological conditions where haemodynamic load, healthy ECM stiffness, and laminar shear stress and sensed by integrins, ion channel, cytoskeletal networks, and intercalated discs, leading to balanced signaling and healthy mitochondrial adaptation. The bottom panel (B) shows pathological remodeling under pressure overload, fibrotic ECM stiffening, and disturbed flow resulting in dysregulated mechanosensors and signaling cascades that drive mitochondrial fragmentation, membrane potential collapse, calcium overload, and oxidative stress‐contributing to cardiac disease progression.

The interplay between mechanical stress and mitochondrial dynamics is not only fundamental to cardiac physiology but also central to the pathogenesis of cardiovascular diseases. Conditions such as cardiac hypertrophy, IRI, and heart failure are characterized by altered mechanical environments that disrupt mitochondrial structure, function, and signaling. These changes contribute to energetic failure, oxidative stress, and maladaptive remodeling, reinforcing the concept that mitochondrial dysfunction is both a consequence and a driver of biomechanical stress (**Figure** **2**).

### 9.1 Cardiac Hypertrophy

Cardiac hypertrophy is a compensatory response to chronic mechanical overload, such as that induced by hypertension or aortic stenosis. While initially adaptive, prolonged hypertrophy leads to structural remodeling, metabolic reprogramming, and mitochondrial dysfunction. Mitochondria, as central regulators of energy production and redox balance, are particularly vulnerable to biomechanical stress, and their maladaptation contributes to the progression from compensated hypertrophy to heart failure. This includes changes to mitochondrial shape ‐ PKM1, a glycolytic enzyme with non‐canonical roles in mitochondrial integrity, is downregulated under pressure overload, leading to mitochondrial fragmentation and exacerbated remodeling.^[^ ^113^ ^]^ Moreover, mitochondrial dysfunction in hypertrophy is associated with altered calcium handling. Cytoplasmic Ca^2^⁺ peaks in neonatal cardiomyocytes directly modulate mitochondrial Ca^2^⁺ uptake via the MCU, highlighting a dynamic buffering role of mitochondria in response to cytosolic calcium transients.^[^ ^114^ ^]^

Mitochondrial remodeling under pressure overload also involves transcriptional regulation (e.g., through YAP1 as discussed above^[^ ^64^ ^]^), metabolic reprogramming, and quality control mechanisms, which play a protective role. PINK1‐mediated mitophagy suppresses the release of mtDNA, which otherwise activates the cGAS–STING inflammatory pathway. This mitophagic response attenuates hypertrophy and preserves mitochondrial function.^[^ ^17^ ^]^

Mechanical load initially triggers adaptive mitochondrial responses, including upregulation of biogenesis genes (PGC1‐α, TFAM) and enhanced ATP production to meet increased energetic demand.^[^ ^14^ ^]^ However, sustained overload progressively shifts this balance toward maladaptation. In advanced stages of dilated cardiomyopathy, mitochondrial fragmentation, impaired oxidative phosphorylation, and metabolic reprogramming lead to ATP depletion and energetic failure.^[^ ^76^ ^]^ This transition from mechanical stress‐induced metabolic enhancement to dysfunction underscores the temporal complexity of mechanosensitive mitochondrial regulation and highlights a critical therapeutic window to preserve mitochondrial integrity during disease progression.

Therapeutic strategies targeting mitochondrial metabolism show promise in mitigating hypertrophic remodeling. Activation of ERRs using SLU‐PP compounds enhances mitochondrial respiration and fatty acid oxidation, improving cardiac function in pressure overload models.^[^ ^76^ ^]^ These findings suggest that restoring mitochondrial integrity and metabolic flexibility may be key to preventing the transition from hypertrophy to heart failure.

### 9.2 Ischemia Reperfusion Injury

IRI occurs when blood supply returns to the heart after a period of ischemia, triggering a cascade of metabolic and mechanical stress responses. While reperfusion is essential for tissue survival, it paradoxically exacerbates cellular damage through mitochondrial dysfunction, oxidative stress, and calcium overload.^[^ ^115^ ^]^ Mitochondria are central to this process, acting as both sensors and amplifiers of injury.^[^ ^4^ ^]^

During IRI, the sudden restoration of oxygen leads to a surge in ROS production, primarily at complexes I and III of the electron transport chain.^[^ ^83^, ^116^ ^]^ This oxidative burst damages mitochondrial membranes, proteins, and DNA, impairing ATP synthesis and promoting apoptosis. Simultaneously, mechanical strain from reperfusion activates MAPK–p38 and RhoA–ROCK pathways, which phosphorylate DRP1 at Ser616, promoting mitochondrial fission and fragmentation.^[^ ^51^, ^52^, ^54^, ^117^ ^]^

Calcium overload is another hallmark of IRI. Reperfusion‐induced calcium influx via stretch‐activated ion channels overwhelms mitochondrial buffering capacity. Excessive calcium uptake through the MCU complex triggers mPTP opening, leading to membrane depolarization and cell death.^[^ ^82^, ^118^, ^119^ ^]^ These events disrupt cristae architecture and compromise oxidative phosphorylation.^[^ ^120^ ^]^ Recent studies have identified mitochondrial CaMKII as a key mediator of metabolic dysfunction in IRI. Activation of mitochondrial calcium/calmodulin‐dependent protein kinase II (CaMKII) impairs oxidative metabolism and promotes dilated cardiomyopathy. Inhibition of this pathway preserves mitochondrial energetics and protects against left ventricular dilation following myocardial infarction.^[^ ^121^ ^]^

Mitochondrial quality control mechanisms such as PINK1‐mediated mitophagy attempt to limit damage by removing dysfunctional mitochondria and suppressing mtDNA‐triggered inflammation.^[^ ^17^ ^]^ However, in severe IRI, these protective responses are often overwhelmed.

Targeting mitochondrial dynamics and metabolism offers promising avenues for cardioprotection. Inhibiting DRP1 activation or enhancing fusion proteins like MFN1/2 can preserve mitochondrial morphology and reduce apoptosis.^[^ ^4^ ^]^ Agents that stabilize mitochondrial membrane potential or block mPTP opening, such as cyclosporine A or chloride depletion have shown efficacy in experimental models.^[^ ^122^ ^]^ Modulating calcium signaling is another strategy. Enhancing MCU gatekeeping via MCU1/2/3 subunits can prevent calcium overload and preserve mitochondrial integrity.^[^ ^119^ ^]^ Additionally, mechanical conditioning techniques such as ischemic preconditioning or low‐shear perfusion have demonstrated protective effects by modulating mitochondrial responses.^[^ ^28^, ^123^ ^]^

### 9.3 Heart Failure

Heart failure (HF) is a progressive syndrome characterized by impaired cardiac output and energy deficiency. Mitochondrial dysfunction is a hallmark of HF, contributing to contractile failure, arrhythmogenesis, and maladaptive remodeling. As the primary source of ATP in cardiomyocytes, mitochondria are central to maintaining cardiac function, and their dysregulation under chronic mechanical and metabolic stress plays a pivotal role in HF pathogenesis.

In HF, mitochondrial dysfunction manifests as impaired oxidative phosphorylation, increased ROS production, calcium dysregulation, and altered mitochondrial dynamics. Chronic biomechanical stress including pressure overload and volume expansion activates maladaptive signaling pathways including MAPK, RhoA–ROCK, and YAP/TAZ, which converge on mitochondrial targets to promote fragmentation, depolarization, and apoptosis.^[^ ^15^, ^51^, ^54^ ^]^

A key feature of failing hearts is the shift from fatty acid oxidation to glycolysis, which reduces ATP yield and contributes to energy starvation. This metabolic reprogramming is accompanied by downregulation of PGC‐1α and ERRs, impairing mitochondrial biogenesis and respiratory capacity.^[^ ^76^ ^]^ Additionally, mitochondrial calcium overload and mPTP opening lead to membrane potential collapse and release of pro‐apoptotic factors such as cytochrome c, exacerbating cardiomyocyte loss.^[^ ^122^ ^]^ In vitro mechanical stress models that mimic features of DCM have shown that cyclic stretch induces mitochondrial fragmentation, ROS accumulation, and membrane depolarization, mimicking the mitochondrial phenotype observed in failing hearts.^[^ ^124^ ^]^ These findings underscore the role of mechanical stress in driving mitochondrial dysfunction and cell death in HF.

Therapeutic approaches targeting mitochondrial dysfunction in HF aim to restore energy balance, reduce oxidative stress, and preserve mitochondrial integrity. Activation of ERRs has been shown to enhance fatty acid oxidation and improve cardiac function in pressure overload models.^[^ ^76^ ^]^ Similarly, stabilizing mitochondrial membrane potential and preventing mPTP opening can mitigate cell death and preserve contractile performance.^[^ ^122^ ^]^ Modulating mitochondrial dynamics is another promising strategy. Inhibiting DRP1‐mediated fission or enhancing fusion via MFN1/2 can restore mitochondrial morphology and function.^[^ ^4^ ^]^ Enhancing mitophagy through PINK1 activation may also help clear damaged mitochondria and reduce inflammation.^[^ ^17^ ^]^ Emerging therapies also include MCU modulators to prevent calcium overload, antioxidants such as MitoTEMPO to scavenge ROS, and cytoskeletal stabilizers to maintain mitochondrial positioning and bioenergetic efficienc.^[^ ^30^, ^89^, ^119^ ^]^ These interventions highlight the therapeutic potential of targeting mitochondrial pathways to halt or reverse HF progression.

## 10 Knowledge Gaps and Future Directions

The intricate relationship between mechanical stress and mitochondrial function in cardiomyocytes has emerged as a critical axis in cardiovascular physiology and pathology. While recent advances have illuminated key mechanotransduction pathways and mitochondrial responses, several unresolved questions and limitations persist. Addressing these knowledge gaps is essential for translating mechanobiological insights into effective therapies for cardiac disease.

### 10.1 Mechanistic Integration Across Scales

One of the most pressing challenges is the lack of integrative models that connect molecular events to whole‐organ outcomes. Most studies focus on isolated pathways in isolated cell systems, such as DRP1‐mediated fission or MCU‐regulated calcium uptake in isolated cardiomyocytes. This system does not account for the for the broader biomechanical context. The heart functions as a multiscale system, where mechanical forces propagate from ECM to cytoskeleton, organelles, and gene expression and multiscale approaches and integrated platforms are needed to bridge molecular mechanosensing with tissue‐level outcomes.^[^ ^125^ ^]^ Developing computational frameworks that simulate mitochondrial responses to mechanical stimuli across spatial and temporal scales could further help predict disease progression and therapeutic efficacy.

### 10.2 Temporal Dynamics and Disease Progression

Most experimental studies capture mitochondrial behavior at discrete time points, often under acute stress conditions. However, cardiac diseases such as hypertrophy and heart failure evolve over weeks to months, involving dynamic shifts in mitochondrial morphology, metabolism, and signaling. Therefore, there is a need for chronic, long‐term studies that track mitochondrial adaptation from early compensatory phases to decompensated states. Time‐resolved imaging, transcriptomics, and metabolomics could reveal critical transitions and therapeutic windows. Moreover, understanding how mechanical stress influences mitochondrial aging and senescence remains an underexplored area with implications for age‐related cardiac decline.

### 10.3 Crosstalk Between Organelles and Compartments

While mitochondria are central to mechanotransduction, their interactions with other organelles such as the nucleus, ER, and sarcoplasmic reticulum (SR), are increasingly recognized as vital for cellular homeostasis.^[^ ^126^, ^127^ ^]^ Disruption of these contacts has been linked to arrhythmogenesis and contractile dysfunction. Future research should focus on mapping these inter‐organelle networks and identifying how mechanical stress alters their architecture and function.

### 10.4 Loading Modes and Metabolic Reorganization

Different loading modes such as isometric (stress‐dominated), isotonic (strain‐driven), and auxotonic (mixed), likely impose distinct mechanotransduction patterns. Stress‐dominated loading may activate integrin–FAK–RhoA/ROCK signaling,^[^ ^21^, ^22^, ^23^, ^35^, ^36^ ^]^ while strain‐driven deformation could modulate cytoskeletal tension and stretch‐activated ion channels, influencing calcium flux and mitochondrial dynamics.^[^ ^39^, ^40^, ^41^, ^42^ ^]^ Evidence from engineered tissues suggests isotonic loading enhances oxidative metabolism and biogenesis, whereas isometric may favor glycolytic shifts under high tension.^[^ ^14^, ^18^, ^19^, ^20^, ^30^ ^]^ Systematic comparisons are lacking; future studies should couple loading mode‐specific metabolic profiling with mitochondrial imaging to clarify whether stress versus strain dominance drives biogenesis, mitophagy, and ROS signaling.^[^ ^16^, ^17^, ^54^, ^65^, ^66^ ^]^

### 10.5 Therapeutic Translation and Delivery Challenges

Although several mitochondrial‐targeted therapies have shown efficacy in preclinical models, their clinical translation remains limited. Agents such as MitoTEMPO, ERR agonists, and DRP1 inhibitors face challenges related to targeted delivery, bioavailability, and off‐target effects. Moreover, the context‐dependent nature of mitochondrial responses‐ where the same pathway may be protective in one setting and deleterious in another‐ complicates therapeutic design. Innovative strategies such as nanocarrier systems, tissue‐specific promoters, and mechanically responsive drug release platforms may enhance precision and minimize side effects.

### 10.6 Understudied Populations and Conditions

Most mechanobiological studies focus on healthy adult male rodents, limiting generalizability. Sex differences, aging, and comorbidities such as diabetes and obesity may significantly alter mitochondrial responses to mechanical stress. Expanding research to include diverse models and patient‐derived cells is essential for developing inclusive and effective therapies.

### 10.7 Mechanosensitive Ageing Pathways

Ageing is associated with progressive alterations in mitochondrial function, mechanical resilience, and cellular signaling in cardiomyocytes. Mechanical stress during ageing can exacerbate mitochondrial fragmentation, reduce ATP output, and increase oxidative stress.^[^ ^128^ ^]^ Notably, members of the SIRT family, particularly SIRT1 and SIRT3, have emerged as key regulators of mitochondrial quality control and redox balance under biomechanical strain. SIRT1 modulates PGC1‐α activity and mitochondrial biogenesis,^[^ ^129^ ^]^ while SIRT3 deacetylates and stabilizes components of the electron transport chain.^[^ ^130^ ^]^ Age‐related decline in SIRT expression may impair these protective mechanisms, contributing to cardiac dysfunction. Future studies should explore how mechanical cues interact with ageing‐associated pathways to influence mitochondrial adaptation and disease progression.

## 11 Conclusion

The heart is a biomechanically active organ that relies on a continuous interplay between mechanical forces and metabolic function to sustain life. At the center of this interplay are mitochondria which are dynamic organelles that not only generate the energy required for contraction but also respond to mechanical cues through changes in morphology, positioning, and function. Mitochondria are not passive energy suppliers but active participants in sensing and responding to mechanical stress. Their dynamic behavior underlies the heart's ability to adapt to physiological demands and contributes to the pathogenesis of cardiovascular disease when dysregulated. Bridging the fields of mitochondrial biology and cardiac mechanobiology holds great promise for uncovering novel therapeutic targets and advancing our understanding of heart disease.

## Conflict of Interest

The authors declare no conflict of interest.
